# Effects of Platelet Count on Blood Pressure: Evidence from Observational and Genetic Investigations

**DOI:** 10.3390/genes14122233

**Published:** 2023-12-18

**Authors:** Zhen He, Zekai Chen, Martin H. de Borst, Qingying Zhang, Harold Snieder, Chris H. L. Thio, on behalf of the International Consortium of Blood Pressure

**Affiliations:** 1 Department of Epidemiology, University Medical Center Groningen, University of Groningen, 9713 GZ Groningen, The Netherlands; z.he@umcg.nl (Z.H.); z.chen@umcg.nl (Z.C.);; 2 Department of Preventive Medicine, Shantou University Medical College, No. 22, Xinling Road, Shantou 515041, China; qyzhang@stu.edu.cn; 3 Department of Internal Medicine, Division of Nephrology, University Medical Center Groningen, University of Groningen, 9713 GZ Groningen, The Netherlands; m.h.de.borst@umcg.nl

**Keywords:** platelet count, blood pressure, Mendelian randomization

## Abstract

Platelet count has been associated with blood pressure, but whether this association reflects causality remains unclear. To strengthen the evidence, we conducted a traditional observational analysis in the Lifelines Cohort Study (*n* = 167,785), and performed bi-directional Mendelian randomization (MR) with summary GWAS data from the UK Biobank (*n* = 350,475) and the International Consortium of Blood Pressure (ICBP) (*n* = 299,024). Observational analyses showed positive associations between platelet count and blood pressure (OR = 1.12 per SD, 95% CI: 1.10 to 1.14 for hypertension; B = 0.07, 95% CI: 0.07 to 0.08 for SBP; B = 0.07 per SD, 95% CI: 0.06 to 0.07 for DBP). In MR, a genetically predicted higher platelet count was associated with higher SBP (B = 0.02 per SD, 95% CI = 0.00 to 0.04) and DBP (B = 0.03 per SD, 95% CI = 0.01 to 0.05). IVW models and sensitivity analyses of the association between platelet count and DBP were consistent, but not all sensitivity analyses were statistically significant for the platelet count-SBP relation. Our findings indicate that platelet count has modest but significant effects on SBP and DBP, suggesting causality and providing further insight into the pathophysiology of hypertension.

## 1. Introduction

Elevated blood pressure is the single largest contributor to disease burden and mortality worldwide. The number of people suffering from hypertension displays an increasing trend and it causes more than 9 million deaths each year globally [1]. The factors protecting against blood pressure elevation include a low-salt diet, increased intake of fruit and vegetables, weight loss, physical exercise, and alcohol abstinence [1]. The etiology of hypertension is complex and not yet fully understood, and more research exploring its determinants is warranted.

A potential determinant for hypertension is platelet count. Platelets have a non-nucleated, disk-like cytoplasmic body, and are produced by megakaryocytes in humans. As a constituent of blood, they can terminate bleeding at the injured vascular site through clot formation, and play a role in immune surveillance [2]. Previously, platelet count was found to be associated with blood pressure and hypertension in both traditional observational [3] and one-sample MR studies [4]. However, these studies were carried out in relatively small samples. Additionally, one-sample MR is susceptible to weak instrument bias in the direction of observational estimates, which in turn are potentially biased due to unobserved confounding. Finally, these studies were carried out in East Asians, and their generalizability to other ethnicities is uncertain. Hence, to strengthen the evidence for causality, validation using large-scale, comprehensively phenotyped biobank data is necessary, as well as two-sample MR methods less susceptible to weak instrument bias [5], in samples of ethnicity other than East Asian.

In this study, we thus aimed to assess a potential causal effect of platelet count on blood pressure by employing two complementary approaches (Supplementary Figure S1) in a triangulation framework [6]: multivariable cross-sectional analyses of the Lifelines Cohort study (*n* = 167,785), and two-sample Mendelian randomization (MR) analysis using genetic instruments to minimize confounding in data from the UK Biobank (UKB, *n* = 458,577 Europeans) and the International Consortium of Blood Pressure (ICBP, *n* = 299,024 Europeans) [7,8]. These approaches are complementary in their key sources of bias, i.e., residual confounding in traditional regression, and horizontal pleiotropy in MR.

### Plain Language Summary

Platelets are a blood component primarily involved in blood clotting. An elevated platelet count has been linked to hypertension, but it is unclear whether this reflects a true causal relationship. We investigated this relation in community-based cohort data from the Lifelines Cohort Study and Biobank. Here, we found a modest but robust association between elevated platelet count and higher blood pressure. Using genetic data from UK Biobank and the International Consortium of Blood Pressure, we aimed to further strengthen the evidence. We did so by conducting Mendelian randomization, a method that exploits the random allocation of genetic variants as a natural experiment. Mendelian randomization corroborated the findings from Lifelines: we found genetic evidence that elevated platelet count modestly increases diastolic blood pressure and, to a lesser extent, systolic blood pressure. A reverse effect was not evident from our analyses: higher blood pressure seems to be, at least in part, a consequence of elevated platelet count and not vice versa. The present study is by far the largest on the relation between platelets and blood pressure. Taking into account the limitations of our study, we conclude that there is a modest potentially causal effect of platelets on blood pressure. Future experimental work is needed to more definitively establish causality, and to assess whether platelets could be a target in the prevention, early diagnosis, and/or treatment of hypertension.

## 2. Methods

### 2.1. Observational Analysis

Cohort profiles describing the Lifelines Cohort Study design and data collection have been published previously [9,10]. A brief description of the Lifelines Cohort can be found in Appendix A. All subjects provided written informed consent prior to data collection. This study complied with the Declaration of Helsinki and was approved by the medical ethical committee (number 2007/152) of the University Medical Center Groningen, The Netherlands.

We constructed a directed acyclic graph (DAG) [11] based on the existing literature (Supplementary Figure S2), from which we identified a minimally sufficient adjustment set (MSAS), consisting of glycated hemoglobin, age, sex, moderate-to-vigorous-intensity physical activity (MVPA), and smoking at baseline (see also Appendix B). From the complete Lifelines data (*n* = 152,728 adults ≥18 years of age), we included 110,117 participants with complete data on blood pressure, platelet count, and MSAS (see flowchart, Supplementary Figure S1). 

Continuous variables were described as mean ± standard deviation (SD) or median (interquartile range [IQR]), and categorical variables as numbers with percentages. Three models were constructed for each outcome (i.e., hypertension, SBP, and DBP): univariable (model 1), age and sex-adjusted (model 2), and MSAS-adjusted (model 3) (Supplementary Figure S2) [11,12]. We also conducted reverse analyses (i.e., blood pressure as exposure and platelet count as outcome). Logistic and robust linear regression was performed using the *stats* and *robustbase* R packages in R version 4.0.3. A two-sided *p* < 0.05 was considered statistically significant. We followed the STROBE checklist [13] for observational studies (Supplementary Table S1).

### 2.2. Mendelian Randomization Analysis

We applied two-sample MR, which yields causal estimates if its three key assumptions (relevance, exchangeability, and exclusion restriction) are satisfied [14]. We utilized GWAS summary data from the UK Biobank (UKB) and the International Consortium of Blood Pressure (ICBP) [7,8] (Supplementary Table S2). 

Platelet count in the UKB was measured using a Beckman Coulter LH750 Haematology Analyser (Beckman Coulter, Inc., Fullerton, CA, USA) [7]. We identified instrumental variables (IVs) for platelet count (*n* = 350,474) from UKB [7] according to a pre-defined SNP selection procedure (Supplementary Figure S1). IVs are listed in Supplementary Tables S3 and S4. We then extracted IV associations with blood pressure from UKB-ICBP summary GWAS data (*n* = 757,601 Europeans) [7,8]. We also used summary statistics of GWAS blood pressure data from UKB (“UKB-only”, BMI-unadjusted) and ICBP (“ICBP-only”, BMI-adjusted) separately as secondary outcomes to assess the effect of sample overlap [15] and potential collider bias [5,16] caused by BMI adjustment in the UKB-ICBP GWAS data. We further performed multivariable MR to account for potential collider bias by including BMI (Supplementary Table S2) [17]. 

Inverse variance-weighted (IVW) random-effects MR [14] was performed as our main analysis [18]. Cochran’s Q-statistic was used to assess heterogeneity. Pleiotropy robust MR methods (Mendelian randomization-Egger, MR-Egger [19]; weighted median [20]; and MR Pleiotropy RESidual Sum and Outlier, MR-PRESSO [21]) were performed as sensitivity analyses. For univariable MR, we applied Steiger filtering [22], but not for multivariable MR, as Steiger filtering is not well established in this context. We assessed the strength of IVs by calculating F-statistics [15,23]. To aid in the interpretation of results, we standardized blood pressure summary GWAS data using a Z-score transformation. Scatter plots, forest plots, funnel plots, and leave-one-out plots were used for visualization of the results and assessment of potential directional pleiotropy. *TwoSampleMR* and *MRPRESSO* packages in R (version 3.6.2 and version 4.0.3) were used for this MR analysis. *p* < 0.05 (two-sided tests) was considered statistically significant. We followed the STROBE-MR checklist (Supplementary Table S5) [24].

## 3. Results

### 3.1. Observational Results

Overall, 110,117 participants with complete data were enrolled into the cross-sectional analysis (median age 45 [IQR 36–52] years, 41% male). Medians of platelet count, SBP, and DBP were 244 (IQR 211–282) × 10^9^/L, 124 (IQR 115–136) mmHg, and 73 (IQR 67–81) mmHg, respectively. Hypertension prevalence was 25.3% (27,858/110,117). We compared descriptives with the complete adult Lifelines sample (*n* = 152,728) and found similar distributions; bias due to missingness is thus unlikely (Table 1).

In MSAS-adjusted models, a higher platelet count was associated with hypertension (OR = 1.12, 95% CI = 1.10 to 1.14), SBP (B = 0.07, 95% CI = 0.07 to 0.08), and DBP (B = 0.07, 95% CI = 0.06 to 0.07) (all per standard-deviation-higher exposure, Table 2 and Supplementary Table S6).

Reversely, hypertension and blood pressure were positively associated with platelet count (B = 0.08, 95% CI = 0.07 to 0.10 for hypertension; B = 0.09, 95% CI = 0.08 to 0.10 for SBP; B = 0.07, 95% CI = 0.07 to 0.08 for DBP; Supplementary Tables S7 and S8).

### 3.2. MR Results

**Figure 1:**
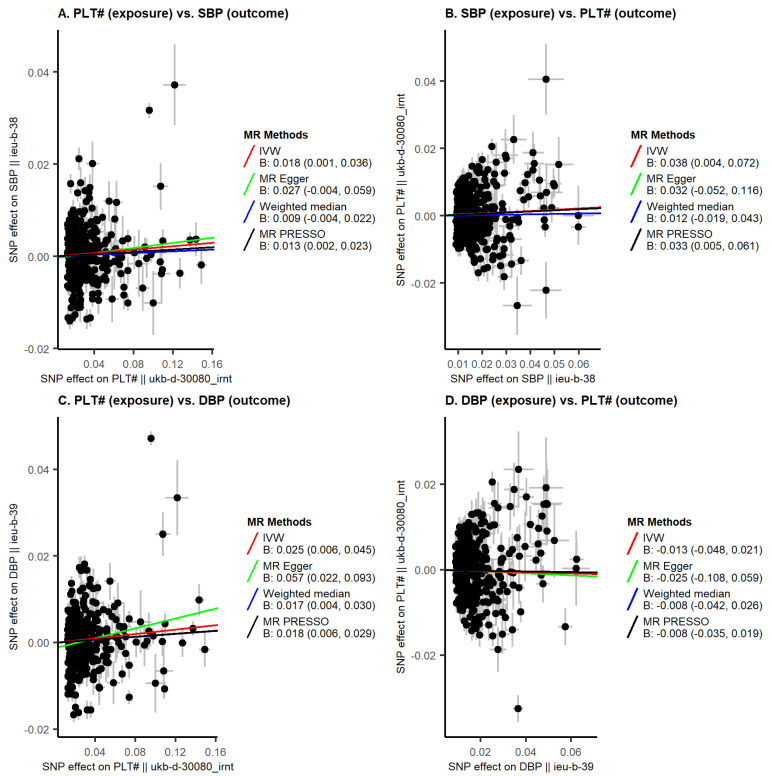
Mendelian randomization scatter plots for platelet count. Panels (**A**,**B**) display bidirectional analyses of the relation between platelet count and SBP. Panels (**C**,**D**) display bidirectional analyses of the relation between platelet count and DBP. The X-axes represent effects on the exposure, while the Y-axes represent effects on the outcome. Each data point represents a single SNP. The slopes of the lines represent effect estimates (standard deviation change in outcome per 1 standard-deviation-higher exposure) from four different MR methods (IVW, MR Egger, weighted median, and MR PRESSO). MR PRESSO: Mendelian Randomization Pleiotropy RESidual Sum and Outlier; PLT#, platelet count; SBP, systolic blood pressure; DBP, diastolic blood pressure; IVW, inverse variance weighted.

**Figure 2:**
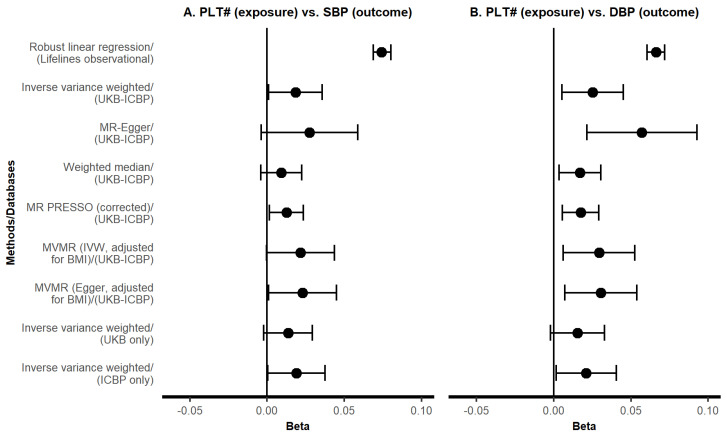
Comparison of effect estimates between observational, Mendelian randomization, and Mendelian randomization sensitivity analyses for systolic/diastolic blood pressure. Panels (**A**,**B**) show the results of forward analyses with SBP and DBP as the outcomes and platelet count as the exposure. The X-axis indicates effect size as standard deviation difference in outcome per one standard-deviation-higher value of exposure. The Y-axis indicates analysis method (with their respective data source). Error bars indicate 95% confidence interval (CI). Lifelines observational regression estimates were adjusted for age, sex, glycated hemoglobin, non-occupational physical activity, and smoking. Abbreviations: MR PRESSO: Mendelian Randomization Pleiotropy RESidual Sum and Outlier; MVMR: multivariable Mendelian randomization; IVW: inverse variance weighted; UKB: UK Biobank; ICBP: International Consortium of Blood Pressure; PLT#, platelet count; SBP: systolic blood pressure; DBP: diastolic blood pressure.

We identified 443 genetic IVs for PLT, of which 360 were available in SBP GWAS data, and 362 in DBP GWAS data (Supplementary Figure S1). Instrument strength was sufficient (one-sided lower-bound confidence limit of F > 140). Genetically predicted platelet count showed a modest positive association with SBP (IVW B = 0.02 standard deviations, 95% CI: 0.00 to 0.04, per standard deviation in exposure) with heterogeneity (Q = 2735.42, degrees of freedom = 359, *p*-value < 0.001) but without evidence of unbalanced pleiotropy (MR-Egger intercept *p*-value = 0.494). Estimates from IVW in the UKB-only and ICBP-only samples, and the various sensitivity analyses (including MR-Egger, weighted median, MR-PRESSO and BMI-adjusted MVMR models), showed similar magnitudes of effects (Figure 1A and Figure 2A, Supplementary Table S9).

The genetically predicted platelet count associated with DBP (IVW B = 0.03, 95% CI: 0.01 to 0.05, UKB-ICBP), which was supported by sensitivity analyses (Figure 1C and Figure 2B, Supplementary Table S9). There was evidence for unbalanced pleiotropy (MR-Egger intercept *p*-value = 0.036) in the platelet count-DBP analysis (Supplementary Table S9), but the MR-Egger estimate was even larger (B = 0.06, 95% CI: 0.02 to 0.09). Multivariable IVW and MR-Egger MR models, accounting for potential collider bias, and the combined effect of collider bias and unbalanced pleiotropy, respectively, yielded consistent estimates of association between platelet count and DBP (MVMR-IVW B = 0.03, 95% CI: 0.01 to 0.05; MVMR-Egger B = 0.03, 95% CI: 0.01 to 0.05, Figure 2B, Supplementary Table S9). Cochran’s Q test indicated heterogeneity in all analyses (all *p*-values < 0.05). Potential directional pleiotropy was explored via forest plots, funnel plots, and leave-one-out plots (Supplementary Figures S3–S6). Reverse MR analyses (i.e., effects of SBP/DBP on platelet count) were non-significant (Figure 1B,D, Supplementary Figure S7 and Table S9).

## 4. Discussion

Using MR, we found evidence for modest but consistent unidirectional positive effects of platelet count on SBP and DBP, supported by not only a wide range of sensitivity analysis, but also large-scale observational analyses. 

A recent one-sample MR study in a Chinese population from Taiwan (*n* = 15,996) showed that platelet count was positively associated with hypertension (β: 0.12; 95% CI: 0.00 to 0.24; *p* = 0.049), while no significant causal effect of hypertension on platelet count was observed in a reverse MR analysis (β: 0.34; 95% CI: −0.16 to 0.85; *p* = 0.185) [4]. Our data corroborate these findings in Europeans, strengthening the overall evidence. Xu et al. [25], in a recent two-sample MR study using largely the same European GWAS data, confirmed the positive effects of platelet count on both SBP and DBP that we observed. However, they also found an effect in the reverse direction of SBP affecting platelet count. Our reverse MR analyses did not yield robust evidence to support the impact of SBP on platelet count. A possible reason for this difference is that our instrument set differed as we applied Steiger filtering, used proxy SNPs, and aligned rather than excluded non-ambiguous palindromic SNPs.

The nature of the mechanism behind the positive association between platelet count and blood pressure is complex and incompletely understood. Potentially, activated platelets enhance the concentration of intracellular calcium ions in vascular smooth muscle cells and further provoke vasoconstriction and a catecholamine response [26], thereby increasing blood pressure. Reactive oxygen species (ROS) can be generated by activated platelets [26,27]. The produced ROS might suppress nitric oxide (NO) bioactivation [28], a substance with a hypotensive effect [29].

Our findings suggest potential causal effects of platelets on blood pressure. Future experimental study is needed to corroborate these findings, and to assess whether intervention on platelet count is safe and clinically meaningful in the prevention or treatment of hypertension and its sequelae. If not a suitable intervention target, alternatively, platelet count could help identify those at increased risk of hypertension.

This study has several strengths. Our observational study is by far the largest on this topic, and uses a comprehensive range of covariates, allowing us to explore the optimal set of confounders. We applied complementary cross-sectional and MR analyses, which increases the credibility of our findings. However, there are several potential shortcomings. First, the DAG may be subject to error in the absence of high-grade evidence (e.g., meta-analysis and/or experimental studies). Second, sample overlap might compound weak instrument bias, although in our study the F statistics were high [23] and sensitivity analyses with varying degrees of sample overlap yielded consistent results. Third, MR is thought to estimate a lifetime effect of platelet count on blood pressure rather than an acute treatment effect. Fourth, we only focused on platelet count and did not consider other platelet indices such as plateletcrit, mean platelet volume, and platelet distribution width. Fifth, our observational findings in the Lifelines Cohort Study are based on a relatively young sample, which may not be generalizable to older populations. Finally, associations from MR can only be considered as causal effects under the key assumptions; functional, experimental work is needed to definitively establish causality.

## 5. Conclusions

Our study demonstrated consistent but modest causal relationships between platelet count and blood pressure. These findings provide insights into the etiology of hypertension, but future research should further investigate the precise mechanisms.

## Figures and Tables

**Table 1:** Participant characteristics of the Lifelines Cohort Study.

		Level	Adult Data (*n* = 152, 728)	Final Sample with Complete Data (*n* = 110, 117)
Sociodemographic	Age, y		44.00 [36.00, 52.00]	45.00 [36.00, 52.00]
	Sex, *n* (%)	Female	89,340 (58.5)	65,200 (59.2)
		Male	63,388 (41.5)	44,917 (40.8)
	Marital status, *n* (%)	In a relationship	113,784 (85.1)	81,834 (85.8)
		Not in a relationship	19,918 (14.9)	13,538 (14.2)
	Education, *n* (%)	No college degree	102,533 (68.6)	72,426 (67.0)
		College degree or higher	46,863 (31.4)	35,642 (33.0)
	Ethnicity, *n* (%)	European	120,486 (98.0)	91,322 (98.1)
		Non-European	2481 (2.0)	1805 (1.9)
Anthropometrics	Weight, kg		78.00 [68.50, 89.00]	78.00 [68.50, 89.00]
	Height, cm		174.79 ± 9.43	174.85 ± 9.37
	BMI, kg/m^2^		25.40 [23.10, 28.30]	25.40 [23.10, 28.20]
Lifestyle	Non-occupational MVPA, minutes/week		185.00 [60.00, 365.00]	186.00 [60.00, 370.00]
	Smoking, *n* (%)	Never smoker	67,586 (46.2)	51,469 (46.7)
		Ex-smoker	48,319 (33.1)	36,357 (33.0)
		Current smoker	30,264 (20.7)	22,291 (20.2)
Blood biomarkers	Platelet count, 10^9^/L		245.00 [211.00, 282.00]	244.00 [211.00, 282.00]
	eGFR, mL/min/1.73 m^2^		96.17 ± 15.07	95.94 ± 15.13
	HbA1c, mmol/mol		37.00 [35.00, 39.00]	37.00 [34.00,39.00]
Outcomes	SBP, mm Hg		125.00 [115.00, 137.00]	124.00 [115.00, 136.00]
	DBP, mm Hg		74.00 [67.00, 81.00]	73.00 [67.00, 81.00]
	Hypertension, *n* (%)	No	111,701 (73.8)	82,259 (74.7)
		Yes	39,646 (26.2)	27,858 (25.3)

Data are presented as number (%), mean ± standard deviation, or in case of non-normal distributions, as median [interquartile range]. eGFR, estimated glomerular filtration rate; SBP, systolic blood pressure; DBP, diastolic blood pressure; *n*, sample size; MVPA, moderate–vigorous physical activity; HbA1c, glycated hemoglobin.

**Table 2:** Results from multivariable logistic and robust linear regression for platelet count as exposure in the Lifelines Cohort Study.

	Logistic Regression (HTN as Outcome)				Robust Linear Regression (SBP as Outcome)				Robust Linear Regression (DBP as Outcome)			
	95% CI				95% CI				95% CI			
Adjustments	OR *	Lower	Upper	*p*	β #	Lower	Upper	*p*	β #	Lower	Upper	*p*
PLT# as exposure (*n* = 110, 117)												
Crude	0.977	0.963	0.990	0.001	−0.019	−0.025	−0.013	<0.001	−0.020	−0.026	−0.014	<0.001
Age + gender	1.147	1.129	1.165	<0.001	0.085	0.079	0.091	<0.001	0.073	0.067	0.078	<0.001
MSAS	1.117	1.099	1.135	<0.001	0.074	0.069	0.080	<0.001	0.066	0.060	0.072	<0.001

* Odds ratio per standard-deviation-higher exposure; # standard deviation difference per one standard-deviation-higher exposure; MSAS included age, glycated hemoglobin, gender, moderate–vigorous physical activity and smoking; PLT#, platelet count; HTN, hypertension; SBP, systolic blood pressure; DBP, diastolic blood pressure; CI, confidence interval.

## Data Availability

The Lifelines Cohort data can be applied for through the following link: http://wiki-lifelines.web.rug.nl/doku.php?id=start (accessed on 1 December 2023). UKB and UKB-ICBP GWAS summary data are publicly available: https://gwas.mrcieu.ac.uk/ (accessed on 1 December 2023). ICBP summary data can be applied for by contacting Mark Caulfield (m.j.caulfield@qmul.ac.uk) or Paul Elliott (p.elliott@imperial.ac.uk).

## Supplementary Materials

The following supporting information can be downloaded at: https://www.mdpi.com/article/10.3390/genes14122233/s1. Figure S1. Flowchart of observational and two-sample Mendelian randomization analyses; Figure S2. Directed acyclic graph; Figure S3. Mendelian randomization sensitivity analysis plots (effect of platelet count on systolic blood pressure); Figure S4. Mendelian randomization sensitivity analysis plots (effect of systolic blood pressure on platelet count); Figure S5. Mendelian randomization sensitivity analysis plots (effect of platelet count on diastolic blood pressure); Figure S6. Mendelian randomization sensitivity analysis plots (effect of diastolic blood pressure on platelet count); Figure S7. Comparison of effect estimates between observational, Mendelian randomization, and Mendelian randomization sensitivity analyses for platelet count. Table S1. STROBE Statement—Checklist of items that should be included in reports of current cross-sectional study; Table S2. Genome-wide association studies (GWAS) used in the current study; Table S3. Instrumental variables (IVs) used for PLT# with SBP/DBP as outcomes; Table S4. Instrumental variables (IVs) used for SBP/DBP with platelet count as outcome; Table S5. STROBE-MR Checklist of Recommended Items to Address in Reports of Mendelian Randomization Studies a; Table S6. Results from multivariable logistic and robust linear regressions for platelet count as exposure in the Lifelines Cohort Study; Table S7. Results from reverse robust linear regression analysis for HTN/SBP/DBP as exposure in the Lifelines Cohort Study; Table S8. Results from reverse robust linear regression for HTN/SBP/DBP as exposure in the Lifelines Cohort Study; Table S9. Observational and MR estimates of the effect of platelet count on blood pressure.

## Appendix A. A Brief Description of the Lifelines Cohort

Lifelines is a multi-disciplinary prospective population-based cohort study examining in a unique three-generation design for the health and health-related behaviours of more than 167,000 persons living in the North of the Netherlands. It employs a broad range of investigative procedures in assessing the biomedical, socio-demographic, behavioural, physical and psychological factors which contribute to the health and disease of the general population, with a special focus on multi-morbidity and complex genetics.

## Appendix B. Description of the Variables used in the Lifelines Study

Platelet count and glycated hemoglobin: measured by Sysmex XE-2100 analyzer (Sysmex Corporation, Kobe, Japan);

Blood pressure: mean of last three out of ten measurements in seated position using an automatic blood pressure monitor (DinaMap, PRO 100V2), adjusted by adding 15 mm Hg for SBP and 10 mm Hg for DBP respectively for subjects taking antihypertensive drugs;

Sex: Male or female;

Non-occupational physical exercise: moderate-to-vigorous-intensity physical activity (MVPA) in leisure time and commuting domains in minutes per week;

Smoking: never smoker, ex-smoker, current smoker.
